# Clinically Significant Behavior Problems among Young Children 2 Years after the Great East Japan Earthquake

**DOI:** 10.1371/journal.pone.0109342

**Published:** 2014-10-21

**Authors:** Takeo Fujiwara, Junko Yagi, Hiroaki Homma, Hirobumi Mashiko, Keizo Nagao, Makiko Okuyama, for the Great East Japan Earthquake Follow-up for Children Study Team

**Affiliations:** 1 Department of Social Medicine, National Research Institute for Child Health and Development, Tokyo, Japan; 2 Department of Neuropsychiatry, Iwate Medical University, Iwate, Japan; 3 Miyagi Prefectural Comprehensive Children's Center, Miyagi, Japan; 4 Department of Neuropsychiatry, Fukushima Medical University, Fukushima, Japan; 5 Nagao Mental Clinic, Mie, Japan; 6 Department of Psychosocial Medicine, National Center for Child Health and Development, Tokyo, Japan; Chiba University Center for Forensic Mental Health, Japan

## Abstract

**Background:**

On March 11, 2011, a massive undersea earthquake and tsunami struck East Japan. Few studies have investigated the impact of exposure to a natural disaster on preschool children. We investigated the association of trauma experiences during the Great East Japan Earthquake on clinically significant behavior problems among preschool children 2 years after the earthquake.

**Method:**

Participants were children who were exposed to the 2011 disaster at preschool age (affected area, n = 178; unaffected area, n = 82). Data were collected from September 2012 to June 2013 (around 2 years after the earthquake), thus participants were aged 5 to 8 years when assessed. Severe trauma exposures related to the earthquake (e.g., loss of family members) were assessed by interview, and trauma events in the physical environment related to the earthquake (e.g. housing damage), and other trauma exposure before the earthquake, were assessed by questionnaire. Behavior problems were assessed by caregivers using the Child Behavior Checklist (CBCL), which encompasses internalizing, externalizing, and total problems. Children who exceeded clinical cut-off of the CBCL were defined as having clinically significant behavior problems.

**Results:**

Rates of internalizing, externalizing, and total problems in the affected area were 27.7%, 21.2%, and 25.9%, respectively. The rate ratio suggests that children who lost distant relatives or friends were 2.36 times more likely to have internalizing behavior problems (47.6% vs. 20.2%, 95% CI: 1.10–5.07). Other trauma experiences before the earthquake also showed significant positive association with internalizing, externalizing, and total behavior problems, which were not observed in the unaffected area.

**Conclusions:**

One in four children still had behavior problems even 2 years after the Great East Japan Earthquake. Children who had other trauma experiences before the earthquake were more likely to have behavior problems. These data will be useful for developing future interventions in child mental health after a natural disaster.

## Introduction

On March 11, 2011, a massive undersea earthquake and subsequent tsunami struck East Japan. With a Richter-scale magnitude of 9.0, the Great East Japan Earthquake was one of the most powerful earthquakes on record and the largest to hit Japan. As of September 2013, the Fire and Disaster Management Agency reported 18,703 deaths, 2,674 missing, and 6,220 injured as a result of the disaster [1]. The most severely affected area was located on the Pacific Ocean side of northeast Japan, encompassing Iwate, Miyagi, and Fukushima prefectures, with 21,262 casualties (18,592 deaths and 2,670 people missing) [1]. Furthermore, 1,706 children lost a parent in the disaster [2].

Several studies have reported the impact of natural disasters on children's mental health, including studies on the 1995 Hanshin-Awaji earthquake in Japan [3]–[5], the 1999 Marmara earthquake in Turkey [6]–[11], the 2004 Indian Ocean earthquake and tsunami [12]–[17], Hurricane Katrina in the USA in 2005 [18]–[21], and the 2008 Sichuan earthquake in China [22]–[25]. Interestingly, most of these previous studies focused on posttraumatic stress disorder (PTSD) or depression as mental health outcomes among children, and few studies investigated the impact of exposure to natural disasters on behavior problems, especially in the preschool to pre-adolescent age range. When assessing mental health status among young children after a natural disaster, it is difficult to diagnose PTSD or depression because young children's responses might be unreliable during the psychiatric interview. Instead, a caregiver's assessment of behavior using the Child Behavior Checklist (CBCL) is better suited to identify young children who need mental health services after a natural disaster, as the CBCL has valid cut-off scores that identify clinically significant behavior problems. It has been suggested that PTSD symptoms can be estimated by CBCL [26], but the validity is arguable [27].

McLaughlin et al. reported that 2 years after Hurricane Katrina approximately 15% of children aged 4 to 17 years showed serious emotional disturbances using the Strength and Difficulties Questionnaire, that is, emotional and behavioral problems that cause significant impairment in role functioning [21]. Further, 3 years after the disaster Lowe et al. assessed behavior problems using the Behavioral Problems Index, which is based on the CBCL, and reported that hurricane-related stressors were indirectly associated with behavior problems [28]. Chemtob et al. assessed the impact of the World Trade Center attack on the mental health of preschool children using the CBCL, and reported that 15–30% of preschool children who were exposed to high-intensity traumatic events related to the World Trade Center attack, such as witnessing the towers collapse, had behavioral symptoms [29].

Moreover, as young children are exposed to multiple trauma experiences after a natural disaster, such as losing a home, parent or friend; witnessing a tsunami or fire; seeing a dead body, or experiencing restrictions on their lifestyle due to radiation, it remains unclear which exposure is associated with which mental disorder or behavior problem. Thienkrua et al. reported that after the tsunami in Sumatra, extreme panic or fear was associated with PTSD, whereas believing that one's own or a family member's life had been in danger was associated with depression in children aged 7 to 14 years in Thailand [12]. To the best of our knowledge, no study has reported the association between specific trauma experiences in a natural disaster and behavior problems among preschool children. Further, there is a need to investigate whether trauma experiences that occurred before the Great East Japan Earthquake, such as the loss of a family member or separation from a caregiver, are associated with behavior problems among children in the affected area, which may have no association in the unaffected area.

Thus, the purpose of this study is to investigate the association of trauma experiences among preschool children on clinically significant behavior problems 2 years after the Great East Japan Earthquake.

## Methods

### Sample

**Figure 1:**
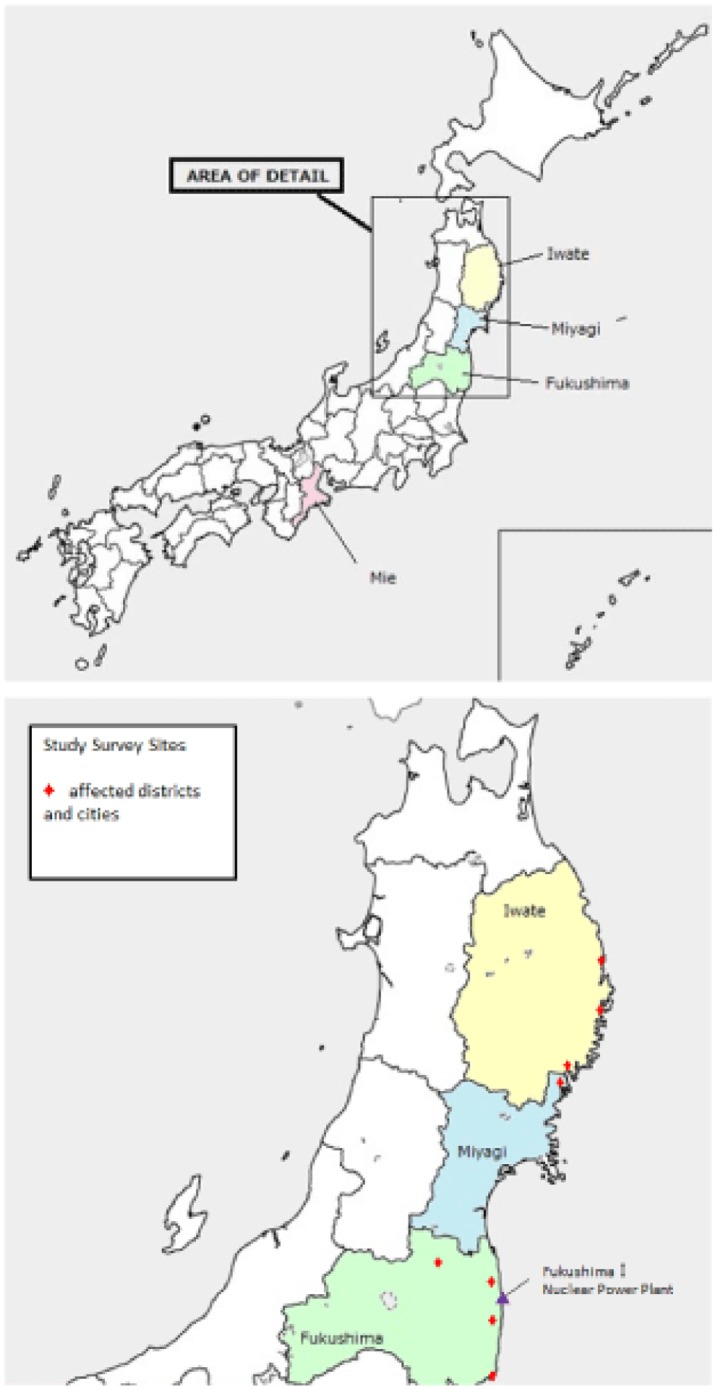
Study sites and provinces affected by the Great East Japan Earthquake.

We recruited affected children with a multistage sampling method in Iwate, Miyagi, and Fukushima prefectures, which were closest to the earthquake epicenter and affected by the tsunami (Figure 1). First, we selected municipalities within each prefecture that were severely affected by the tsunami (coastal side) and radiation caused by the nuclear power plant explosion in Fukushima prefecture. Second, we invited preschools in the selected municipalities to participate. In Iwate prefecture, three municipalities were selected, and four of 32 preschools agreed to participate. In Miyagi prefecture, one municipality was selected, and two of 16 preschools agreed to be involved. Further, in Fukushima prefecture, four municipalities were selected and four of 120 preschools agreed to participate. Third, we defined our target sample as children who were enrolled in a class of 3- to 5-year-olds in the fiscal year of 2010, that is, children who experienced the earthquake on March 11, 2011. Then, from September 2012 to June 2013, principals or staff of the preschools asked the caregivers of the targeted children (N  =  787) to participate in the study. Finally, the caregivers of 205 children gave informed consent for their child to participate (consent rate: 26.0% of target children) and 178 children (Iwate, 59, Miyagi, 53, Fukushima, 66) completed the questionnaire or interview (participation rate: 87.3% of consented children; 170 completed the questionnaire (95.5%) and 150 completed the interview (84.3%)). Our solicitations to participate were largely refused due to the high transience of residents who had to relocate to other areas after the earthquake, especially in Fukushima. Research coordinators obtained written informed consent from all participants. For children, written informed consent was obtained from the child's parent or legal guardian. The Research Ethics Committee at the National Center for Child Health and Development approved this study, including the informed consent procedure.

For comparison, we selected Mie prefecture, which is located in West Japan and was unharmed by the earthquake and tsunami on March 11, 2011. Similar to the sampling strategy in the affected area, two municipalities were selected in Mie prefecture, in which one preschool agreed to participate. Children enrolled in the class of 3- to 5-year-olds in the fiscal year of 2010 were selected and recruited, and 30 out of 220 eligible children participated in the study (consent and participation rate: 13.6%). Two additional communities were selected in the municipalities, and caregiver consent to participate was obtained for 52 out of 608 eligible children (consent and participation rate: 8.6%), resulting in a sample of 82 children from unaffected areas (total consent and participation rate: 9.9%).

### Measurements

Trauma exposure related to the physical environment was assessed via questionnaires administered from September 2012 to June 2013 (around 2 years after the earthquake). Trauma exposure related to the physical environment included status of the home (lost or completely damaged, partially damaged, or not damaged), experience of staying at a shelter immediately after the earthquake, living in temporary housing, evacuating to a relative's house, and family members living in separate places. We assessed these exposures by asking, for example, “Did you stay at a shelter at the time of the Great East Japan Earthquake?”, for which the response items were “yes” or “no”.

Information on severe trauma exposure was collected through interviews by child psychiatrists or clinical psychologists, who were blinded to the children's psychopathological status. In defining trauma exposure, we referred to a previous study assessing children's mental health [12] and the experiences reported in the affected area of a tsunami. Severe trauma exposure assessed by the semi-structured interview method included separation from caregivers, loss of a close family member, loss of distant relatives or friends, witnessing the tsunami waves, witnessing someone being swept away by the tsunami, witnessing a fire, seeing a dead body, hearing the sound of the nuclear power plant explosion, and experiencing restrictions on their lifestyle due to radiation (e.g. unable to play outside, drink tap water, or eat local food).

Caregivers were also asked questions using the Trauma Events Screening Inventory (TESI-C), modified for use with preschool children [29] and further adapted for use in Japan, inquiring whether the child had experienced a wide range of traumatic events, including the experience of a close friend or family member being involved in a serious accident, suffering from a serious illness or dying; self-injury or serious illness; separation from a caregiver; assault, bullying, or other exposure to violence; and exposure to a natural disaster. Response items included “yes”, “no”, or “unknown”, and only “yes” responses were coded as having the actual experience.

Behavior problems were assessed with the CBCL, which targets children aged 4 to 18 years. Ratings were completed by caregivers [30]. The T score of the CBCL internalizing, externalizing, and total problem scores were calculated using standardized distribution among Japanese children, and a T score over 63 was defined as having clinically significant behavior problems [31].

Covariates, including the child's age, sex, number of siblings, parental age, education, and father's occupation were collected via questionnaire.

### Analysis

First, the associations between trauma exposure and behavior problems were analyzed using a bivariate poisson regression model because of the high prevalence of the outcome [32], [33]. Further, multivariate poisson regression using significant variables in bivariate regression was used to examine the independent associations between trauma exposure variables and behavior problems. Moreover, we analyzed the interaction effects between exposure to other trauma before the earthquake and disaster exposure for behavior problems. These analyses were implemented primarily among children in the affected area, and for comparison, the association between other trauma exposure before the earthquake and behavior problems was conducted separately among children in the unaffected area. Stata MP 12 was used for analysis.

## Results

Table 1 shows the demographic characteristics of children and their caregivers in the affected area and the rate of clinically significant behavior problems determined by the CBCL. Children's mean age was around 7 years, sex distribution was equal, and approximately 22% had no siblings. In the unaffected area, caregivers who responded (mostly mothers) were older and more highly educated compared to the affected area. Overall, in the affected area the clinical cut-off was exceeded for internalizing, externalizing, and total behavior problems in 27.7% (95% confidence interval [CI]: 20.9–34.4), 21.2% (95% CI: 15.0–27.4), and 25.9% (95% CI: 19.2–32.5) of participants, respectively. The rate of clinically significant behavior problems did not significantly differ by prefecture, although children in Fukushima showed lower rates.

**Table 1:** Demographic characteristics, trauma exposure related to the Great East Japan Earthquake, other trauma events before the earthquake, and clinically significant behavior problems among young children in the affected area 2 years after the earthquake (N = 170).

Characteristics		n or Mean	% or SD
Mean child age in years		7.1	1.0
Child age group	5 years	23	13.5
	6 years	64	37.7
	7 years	38	22.4
	8 years	45	26.5
Child sex	Boys	84	49.4
	Girls	86	50.6
Number of siblings	No sibling	37	21.8
	1 sibling	83	48.8
	2+ siblings	48	28.2
	Missing	2	1.2
Mean caregiver age in years		36.3	6.2
Caregiver's education	High school or less	79	46.5
	Some college	69	40.6
	College+	20	11.8
	Missing	2	1.2
Father's occupation before earthquake	Manual/Other/Unemployed	88	51.8
	Non-manual	38	22.4
	No response	44	25.9
Exposure to trauma events related to the Great East Japan Earthquake	Home lost or completely damaged	45	26.5
	Home partially damaged	43	25.3
	Stayed at shelter	50	29.4
	Lived in temporary housing	34	20.0
	Evacuated to relative's house	96	56.5
	Family members lived in separate places	53	31.2
	Separation from caregiver	54	31.8
	Lost close family member or relative	11	6.5
	Lost distant relative or friend	21	12.4
	Witnessed tsunami waves	63	37.1
	Witnessed someone being swept away by tsunami	11	6.5
	Witnessed a fire	30	17.7
	Saw a dead body	3	1.8
	Heard the sound of nuclear power plant explosion	3	1.8
	Experienced restrictions on lifestyle due to radiation	32	18.8
	Any of these events	157	92.4
	Number of these events	3.23	1.94
Exposure to other trauma events before the Great East Japan Earthquake	Involvement in a serious accident	5	2.9
	Witnessed a serious accident	0	0
	Attacked by a dog or other animals	0	0
	Had a close friend or family member who had a serious illness	28	16.5
	Death of a close friend or family member	33	19.4
	Visited hospital due to serious disease or injury, or underwent a serious medical procedure, or admitted to hospital	15	8.8
	Separated from a caregiver	27	15.9
	Experienced sexual assault	0	0
	Experienced other criminal assault	3	1.8
	Bullied by peers at preschool or in the neighborhood	3	1.8
	Experienced violence from a close friend or family member	2	1.2
	Witnessed a violent incident involving a close friend or family member	5	2.9
	Had a close friend or family member who attempted suicide	2	1.2
	Experienced a previous natural disaster	1	0.6
	Other stressful events	5	2.9
	Any of these events	78	45.9
	Number of these events	0.74	0.98
CBCL clinically significant behavior problems	Internalizing problems	47	27.7
	Externalizing problems	36	21.2
	Total problems	44	25.9

CBCL: Child Behavior Checklist.

Regarding the distribution of trauma experience, 157 children (92.4%) experienced trauma events related to the Great East Japan Earthquake, in which the mean number of trauma events was 3.23 (SD = 1.94, range 0–9). More specifically, the homes of about half of the participants in the affected area were lost, completely damaged, or partially damaged. In terms of traumatic events revealed in the interviews, witnessing tsunami waves was the most frequent (44%), followed by separation from caregivers (39%), restrictions on their lifestyle due to radiation (28%), witnessing a fire (21%), losing distant relatives or friends (18%), and losing a close family member or relative (10%). Further, 45.9% experienced a traumatic event before the earthquake, in which the mean number of other trauma events before the earthquake was 0.74 (SD = 0.98, range 0–4), mostly the death of a close friend or family member (19.4%), a close friend or family member having a serious illness (16.5%), and being separated from their caregiver (15.9%).

Associations of demographics and traumatic experience with clinically significant internalizing behavior problems are shown in Table 2. In our bivariate model, we found that the deaths of distant relatives or friends and other trauma experienced before the earthquake showed significant association with clinically significant internalizing behavior problems (47.6% vs. 20.2%, rate ratio [RR]: 2.36, 95% CI: 1.10–5.07), and they remained significant even in the multivariate model, which were mutually adjusted. That is, the rate ratio suggests that children who lost distant relatives or friends were 2.22 times (95% CI: 1.03–4.78) more likely to show clinically significant internalizing problems, independent of other trauma experiences before the earthquake. Similarly, children in the affected area who experienced other trauma before the earthquake were 2.22 times (95% CI: 1.22–4.07) more likely to show clinically significant internalizing problems, regardless of trauma exposure related to the earthquake, at 2 years after the earthquake. The interaction effect between experience of other trauma before the earthquake and disaster exposure was not observed (p for interaction term  = 0.502) for internalizing problems, although 28 out of 73 (38.4%) children who experienced both other trauma before the earthquake and trauma related to the earthquake had internalizing problems. This is a higher proportion compared to children who experienced trauma related to the earthquake only and had no prior trauma exposure (15 out of 84, 17.9%, p for chi-square  = 0.004). The association between other trauma experiences and internalizing problems was not found in the unaffected area (RR: 0.64, 95% CI: 0.07–5.73, data not shown).

**Table 2:** Bivariate and multivariate analyses of CBCL clinically significant internalizing behavior problems among young children 2 years after the Great East Japan Earthquake (N = 170).

			CBCL clinically significant internalizing behavior problems							
			n	%		Bivariate RR	95% CI		Multivariate RR	95% CI
Demographics	Child age group	5–6 y	19	21.8		reference				
		7–8 y	28	33.7		1.54	(0.86–2.77)			
	Child sex	Boy	28	33.3		reference				
		Girl	19	22.1		0.66	(0.37–1.19)			
	Number of siblings	No sibling	8	21.6		reference				
		1 sibling	26	31.3		1.45	(0.66–3.20)			
		2+ siblings	12	25.0		1.16	(0.47–2.83)			
	Caregiver age group	36 or less	28	28.6		reference				
		37+	19	26.4		0.92	(0.52–1.65)			
	Education	High school or less	18	22.8		reference				
		Some college+	29	32.6		1.43	(0.79–2.58)			
	Father's occupation before earthquake	Manual/Other/Unemployed	19	21.6		reference				
		Non-manual	12	31.6		1.46	(0.71–3.01)			
		No response	16	36.4		1.68	(0.87–3.28)			
										
Traumatic experience	Status of home	Lost or completely damaged	13	28.9		0.99	(0.50–1.94)			
		Partially damaged	10	23.3		0.79	(0.38–1.66)			
		Not damaged	24	29.3		reference				
	Stayed at shelter	Yes	17	34.0		1.50	(0.81–2.77)			
		No	25	22.7		reference				
	Lived in temporary housing	Yes	10	29.4		1.15	(0.57–2.33)			
		No	33	25.6		reference				
	Evacuated to relative's house	Yes	22	22.9		0.70	(0.39–1.26)			
		No	22	32.8		reference				
	Family members living in separate places	Yes	14	26.4		1.41	(0.76–2.62)			
		No	30	27.5		reference				
	Separation from caregiver	Yes	17	31.5		1.24	(0.65–2.37)			
		No	20	25.3		reference				
	Lost close family member or relative	Yes	2	18.2		0.63	(0.15–2.62)			
		No	31	29.0		reference				
	Lost distant relative or friend	Yes	10	47.6		**2.36**	**(1.10–5.07)**		**2.22**	**(1.03–4.78)**
		No	19	20.2		reference			reference	
	Witnessed tsunami waves	Yes	17	27.0		0.97	(0.51–1.82)			
		No	22	27.9		reference				
	Witnessed someone being swept away by tsunami	Yes	4	36.4		1.36	(0.48–3.83)			
		No	35	26.7		reference				
	Witnessed a fire	Yes	9	30.0		1.10	(0.52–2.32)			
		No	30	27.3		reference				
	Saw a dead body	Yes	2	66.7		2.41	(0.58–10.02)			
		No	37	27.6		reference				
	Heard the sound of nuclear power plant explosion	Yes	0	0.0		NA				
		No	31	25.6						
	Experienced restrictions on lifestyle due to radiation	Yes	8	25.0		0.99	(0.44–2.23)			
		No	21	25.3		reference				
	Exposure to any trauma events or other trauma before the earthquake	Yes	31	39.7		**2.29**	**(1.25–4.18)**		**2.22**	**(1.22–4.07)**
		No	16	17.4		reference				

CBCL: Child Behavior Checklist.

RR: rate ratio.

In our bivariate model, no traumatic experiences related to the earthquake were associated with externalizing problems, while other trauma experiences before the earthquake were significantly associated (Table 3). In the multivariate model adjusted for father's occupation, which showed significant association with externalizing problems in the bivariate model, children in the affected area who experienced other trauma before the earthquake were 2.41 times (95% CI: 1.16–4.99) more likely to show externalizing behavior problems. The interaction effect between experience of other trauma before the earthquake and disaster exposure was not observed (p for interaction term  = 0.296) for externalizing problems, although 24 out of 73 (32.9%) children who experienced both other trauma before the earthquake and trauma related to the earthquake had externalizing problems. This is a higher proportion compared to children exposed to disaster trauma only (9 out of 84 (10.7%), p for chi-square  = 0.001). This association was not found in the unaffected area (RR: 1.28, 95% CI: 0.32–5.12, data not shown).

**Table 3:** Bivariate and multivariate analysis of CBCL clinically significant externalizing behavior problems among young children 2 years after the Great East Japan Earthquake (N = 170).

			CBCL clinically significant externalizing behavior problems							
			n	%		Bivariate RR	95% CI		Multivariate RR	95% CI
Demographics	Child age group	5–6 y	14	16.1		reference				
		7–8 y	22	26.5		1.65	(0.84–3.22)			
	Child sex	Boy	19	22.6		reference				
		Girl	17	19.8		0.87	(0.45–1.68)			
	Number of siblings	No sibling	8	21.6		reference				
		1 sibling	16	19.3		0.89	(0.38–2.08)			
		2+ siblings	11	22.9		1.06	(0.43–2.64)			
	Caregiver age group	36 or less	16	16.3		reference				
		37+	20	27.8		1.70	(0.88–3.28)			
	Education	High school or less	18	22.8		reference				
		Some college+	18	20.2		0.89	(0.46–1.71)			
	Father's occupation before earthquake	Manual/Other/Unemployed	14	15.9		reference			reference	
		Non-manual	7	18.4		1.16	(0.47–2.87)		1.02	(0.41–2.53)
		No response	15	34.1		**2.14**	**(1.03–4.44)**		1.69	(0.80–3.58)
Traumatic experience	Status of home	Lost or completely damaged	10	22.2		1.30	(0.58–2.93)			
		Partially damaged	12	27.9		1.63	(0.76–3.53)			
		Not damaged	14	17.1		reference				
	Stayed at shelter	Yes	12	24.0		1.39	(0.67–2.86)			
		No	19	17.3		reference				
	Lived in temporary housing	Yes	7	20.6		0.98	(0.43–2.26)			
		No	27	20.9		reference				
	Evacuated to relative's house	Yes	16	16.7		0.62	(0.32–1.22)			
		No	18	26.9		reference				
	Family members living in separate places	Yes	10	18.9		0.82	(0.40–1.71)			
		No	25	22.9		reference				
	Separation from caregiver	Yes	12	22.2		1.17	(0.55–2.50)			
		No	15	19.0		reference				
	Lost close family member or relative	Yes	0	0		NA				
		No	26	24.3						
	Lost distant relative or friend	Yes	4	19.1		0.99	(0.34–2.94)			
		No	18	19.2		reference				
	Witnessed tsunami waves	Yes	16	25.4		1.67	(0.79–3.53)			
		No	12	15.2		reference				
	Witnessed someone being swept away by tsunami	Yes	3	27.3		1.43	(0.43–4.73)			
		No	25	19.1		reference				
	Witnessed a fire	Yes	7	23.3		1.28	(0.54–3.03)			
		No	20	18.2		reference				
	Saw a dead body	Yes	0	0		NA				
		No	28	20.9						
	Heard the sound of nuclear power plant explosion	Yes	0	0.0		NA				
		No	23	19.0						
	Experienced restrictions on lifestyle due to radiation	Yes	4	12.5		0.61	(0.21–1.81)			
		No	17	20.5		reference				
	Exposure to any trauma events or other trauma before the earthquake	Yes	25	32.1		2.68	(1.32–5.45)		2.41	(1.16–4.99)
		No	11	12.0		reference			reference	

CBCL: Child Behavior Checklist.

RR: rate ratio.

Finally, the associations of CBCL clinically significant total behavior problems with demographics and traumatic experiences are shown in Table 4. As with the results regarding externalizing problems, no traumatic experiences related to the earthquake were associated with total behavior problems, while other trauma experiences before the earthquake were significantly associated. In the multivariate model adjusted for child age, which showed a marginal association with total behavior problems in the bivariate model, children in the affected area who experienced other trauma before the earthquake were 2.98 times (95% CI: 1.53–5.81) more likely to show total behavior problems. The interaction effect between experience of other trauma before the earthquake and disaster exposure was not observed (p for interaction term  = 0.242) for total behavior problems, although 31 out of 73 (42.5%) children who experienced both other trauma before the earthquake and trauma related to the earthquake had total behavior problems. This is a higher proportion compared to children exposed to disaster trauma only (10 out of 84 (11.9%), p for chi-square <0.001). This association was not found in the unaffected area (RR: 1.03, 95% CI: 0.20–5.28, data not shown).

**Table 4:** Bivariate and multivariate analysis of Child Behavior Checklist (CBCL) clinically significant total behavior problems among young children 2 years after the Great East Japan Earthquake (N = 170).

			CBCL clinically significant total behavior problems							
			n	%		Bivariate PR	95% CI		Multivariate PR	95% CI
Demographics	Child age group	5–6 y	16	18.4		reference			reference	
		7–8 y	28	33.7		1.83	(0.99–3.39)		1.25	(0.93–1.67)
	Child sex	Boy	27	32.1		reference				
		Girl	17	19.8		0.61	(0.34–1.13)			
	Number of siblings	No sibling	13	35.1		reference				
		1 sibling	22	26.5		0.75	(0.38–1.50)			
		2+ siblings	8	16.7		0.47	(0.20–1.14)			
	Caregiver age group	36 or less	23	23.5		reference				
		37+	21	29.2		1.24	(0.69–2.25)			
	Education	High school or less	21	26.6		reference				
		Some college+	23	25.8		0.97	(0.54–1.76)			
	Father's occupation before earthquake	Manual/Other/Unemployed	18	20.5		reference				
		Non-manual	11	29.0		1.42	(0.67–3.00)			
		No response	15	34.1		1.67	(0.84–3.31)			
										
Traumatic experience	Status of home	Lost or completely damaged	12	26.7		1.21	(0.59–2.52)			
		Partially damaged	14	32.6		1.48	(0.74–2.98)			
		Not damaged	18	22.0		reference				
	Stayed at shelter	Yes	14	28.0		1.28	(0.66–2.48)			
		No	24	21.8		reference				
	Lived in temporary housing	Yes	9	26.5		1.03	(0.50–2.16)			
		No	33	25.6		reference				
	Evacuated to relative's house	Yes	22	22.9		0.81	(0.44–1.49)			
		No	19	28.4		reference				
	Family members living in separate places	Yes	15	28.3		1.14	(0.61–2.15)			
		No	27	24.8		reference				
	Separation from caregiver	Yes	17	31.5		1.38	(0.71–2.68)			
		No	18	22.8		reference				
	Lost close family member or relative	Yes	1	9.1		0.31	(0.04–2.30)			
		No	31	29.0		reference				
	Lost distant relative or friend	Yes	8	38.1		1.71	(0.76–3.85)			
		No	21	22.3		reference				
	Witnessed tsunami waves	Yes	19	30.2		1.40	(0.73–2.70)			
		No	17	21.5		reference				
	Witnessed someone being swept away by tsunami	Yes	4	36.4		1.49	(0.53–4.21)			
		No	32	24.4		reference				
	Witnessed a fire	Yes	10	33.3		1.47	(0.70–3.05)			
		No	25	22.7		reference				
	Saw a dead body	Yes	1	33.3		1.28	(0.17–9.32)			
		No	35	26.1		reference				
	Heard the sound of nuclear power plant explosion	Yes	0	0.0		NA				
		No	28	23.1						
	Experienced restrictions on lifestyle due to radiation	Yes	6	18.8		0.78	(0.31–1.94)			
		No	20	24.1		reference				
	Exposure to any trauma events or other trauma before the earthquake	Yes	32	41.0		**3.15**	**(1.62–6.11)**		**2.98**	**(1.53–5.81)**
		No	12	13.0		reference			reference	

CBCL: Child Behavior Checklist.

RR: rate ratio.

## Discussion

We found that clinically significant behavior problems were reported in 26% of young children 2 years after the Great East Japan Earthquake. Interestingly, we observed that the rate of internalizing problems (28%) was higher than the rate of externalizing problems (21%) in the affected area. As internalized problems are less likely to be recognized compared to externalized problems in this age group, these behavior problems may have been underestimated during the 2 years following the earthquake.

To the best of our knowledge, this is the first study to show the rate of behavior problems using the CBCL among 5- to 8-year-old children 2 years after the Great East Japan Earthquake. The rate of clinically significant behavior problems using the CBCL was equivalent to findings from the study of preschool children exposed to the World Trade Center attack, for which the rate was 15–30% for each subscale of the CBCL [29]. McLaughlin et al. reported that 2 years after Hurricane Katrina, 15% of children aged 4 to 17 years had serious emotional disturbances (defined by a combination of scores for conduct problems, hyperactivity-inattention, emotional symptoms, peer problems, and symptom-related impairment measured by the Strengths and Difficulties Questionnaire) [21]. Furthermore, depression, which can be considered an internalizing problem, was reported in 12% of children aged 7 to 14 years who had been living for 9 months in displacement camps after the 2004 Indian Ocean earthquake and tsunami [12]. Although a simple comparison is not plausible because of differences in age, ethnicity, type of disaster, and timing of the assessment after the disaster, it is noteworthy that the rate of children aged 5 to 8 years with behavior problems after the Great East Japan Earthquake was higher than the rate after Hurricane Katrina or after the 2004 Indian Ocean disaster in affected parts of southern Thailand, which illustrates the severity of the trauma experienced by children residing in the affected area of Japan as a result of the earthquake, tsunami, and subsequent radiation crisis.

We found that losing distant relatives or friends was associated with clinically significant internalizing problems, but not externalizing or total behavior problems. This is consistent with previous research, which shows that depression among children after the 2004 Indian Ocean tsunami in devastated areas of southern Thailand was associated with the experience of a close family member or friend being injured [12]. The experience of losing a relative or friend may cause children to feel sadness, fear, or regret because they were unable to help during the tsunami, which leads to internalizing behavior. In our study, the lack of association of depression with losing a close family member or relative might have been due to selection bias and lack of power; that is, caregivers who lost a family member were less likely to participate in this study. In our study, only two children lost a close family member or relative. A previous study also reported that a natural disaster's long-term repercussions on children's mental health is influenced by various determinants including being separated from caregivers, experiencing traumatic events, and feeling that one's life or that of a close friend or family member is under threat [34].

We also found that other trauma experiences that occurred before the earthquake were significant risk factors for behavior problems among young children who were exposed to the earthquake, regardless of internalizing or externalizing behavior problems. This is consistent with findings of behavior problems in children after the World Trade Center attacks that showed a combined effect of other trauma exposure before the attacks, and traumatic events related to the attacks showed synergistic effects on behavior problems. High-risk approaches targeting young children who have been exposed to other trauma before the earthquake might be an efficient strategy to provide mental healthcare resources, which are limited after the earthquake.

Several limitations of this study must be addressed. First, the participants were not a representative sample of the municipalities affected by the earthquake; that is, we selected municipalities where one of the authors had enough personal connections to conduct this study. Furthermore, children with severe mental disorders in the target population may have been reluctant to join this study because they had already received psychiatric services. Alternatively, caregivers who were concerned about their children's mental health might have been more likely to participate in this study. Nonetheless, the significance of this study is that it reveals the rate of children with behavior problems in a community sample. Second, the CBCL was filled out by caregivers only; thus, behavior problems in school were not included. Further research should combine teacher and caregiver CBCL ratings. Third, severe traumatic experiences related to the earthquake were assessed through interviews with children, but some children might not describe their true experiences to an interviewer. We double-checked the reported experiences by interviewing caregivers and preschool teachers, but we considered the trauma described by child participants themselves as being the most authentically representative of the children's trauma. Fourth, our sample size was relatively small; thus, there may have been too few participants to properly assess the significance of the associations between specific traumatic experiences and behavior problems. However, even with this small sample size, we demonstrated that some specific traumatic experiences were associated with behavior problems, which informs suggestions for future prevention of behavior problems after a natural disaster. Fifth, the response rate is not very high, especially in Fukushima. This is because most children were evacuated from the original community as a result of the radiation crisis, thus it was extremely difficult to obtain consent.

In conclusion, clinically significant behavior problems were found in one of four young children living in the area affected by the Great East Japan Earthquake, even 2 years after the disaster. Specific trauma experience, i.e., loss of distant relatives or friends, was associated with internalizing problems, but not externalizing or total behavior problems. Moreover, children who experienced other trauma events before the earthquake were more likely to have behavior problems. Based on these findings, we make a call for further interventions for young children exposed to the disaster, such as psychoeducation programs to provide information on traumatic symptoms, coping strategies and recovery, in collaboration with school and preschool principals, teachers, and school counselors. Moreover, larger studies using representative samples are essential to further address the mental health needs of young children exposed to the 2011 disaster.
